# Comparison of Lower Extremity EMG Muscle Testing With Hands-Free Single Crutch vs Standard Axillary Crutches

**DOI:** 10.1177/2473011420939875

**Published:** 2020-09-02

**Authors:** Cuyler Dewar, Kevin D. Martin

**Affiliations:** 1 1846 Boston University, Boston, MA, USA; 2 Evan Army Community Hospital, Ft Carson, CO, USA

**Keywords:** nonweightbearing, lower extremity injury, standard axillary crutches, hands-free single crutch, iWALKFree, electromyography, assistive device

## Abstract

**Background:**

In order to maintain nonweightbearing restrictions of the lower extremity, an assistive device must be utilized. Currently most devices require the restricted limb to be held in a static position while the contralateral extremity provides forward propulsion. Atrophy and disuse conditions ensue rapidly, slowing healing and prolonging recovery. A hands-free single crutch (HFSC) utilizes both lower extremities, potentially reducing atrophy. The purpose of this study was to examine the electromyographic (EMG) differences between an HFSC and standard axillary crutches (SAC).

**Methods:**

A prospective, crossover study was performed using 21 healthy volunteers from an active duty foot and ankle clinic. Demographic data were obtained and then subjects were fitted with an HFSC and SAC. Wireless surface EMG sensors were applied to the belly of the rectus femoris (RF), vastus lateralis (VL), lateral gastrocnemius (LG), and the gluteus maximus (GM) by a board-certified orthopedic surgeon. Subjects then ambulated at a self-selected velocity for 30 m while 15 seconds of the gait cycle were recorded for each device. Mean muscle activity and the maximum voluntary isometric contraction (MVIC) were recorded.

**Results:**

The RF, GM, and LG showed significantly increased levels of muscle activity while using the HFSC compared to SAC (respectively *P* = .05, *P* = .03, *P* = .03). The VL did not show significantly higher muscle activity while using the HFSC (*P* = .051). The RF, GM, and VL showed statistically significant higher MVIC percentages while using the HFSC compared with SAC (respectively *P* = .005, *P* = .005, *P* = .013). The LG did not show significantly higher MVIC percentage while using the HFSC (*P* = .076).

**Conclusion:**

The HFSC subjects demonstrated increased muscle recruitment and intensity while maintaining cyclic contractions consistent with bipedal gait pattern. SAC demonstrated less recruitment and intensity with an isometric pattern regardless of the phase of gait.

**Clinical Relevance:**

Muscle atrophy following lower extremity immobilization.

## Introduction

Lower extremity injuries occur frequently and impact many different populations including the general public, athletes, and those in the military.^12^ A common location of lower extremity injury is the foot and ankle. One study found that foot and ankle injuries accounted for 27% of musculoskeletal injuries in athletes and 21% of those injuries caused the athlete to miss time from the sport.^16^ Foot and ankle injuries often require a period of nonweightbearing during recovery that can cause significant costs to the patient.^20,30^ They will have a decreased quality of life with limited mobility, possible missed work, and will have medical expenses. Their employer will also be affected. One study found that lower extremity injuries cost the US Army millions of dollars, reduced training efficiency, and detracted from force strength.^12^

Patient’s nonweightbearing timeline depends on many factors, including comorbidity and injury type.^9,30^ In order to maintain nonweightbearing restrictions of the lower extremity, an assistive device must be used. These ambulatory aids function to allow for increased mobility, balance, and independence during recovery.^2^ Ambulatory devices include canes, scooters, and wheelchairs, but the most prescribed ambulatory assistive device for nonweightbearing is axillary crutches.^1,2,13,19,31^ Standard axillary crutches (SACs) are an inexpensive ambulatory aid that provide increased independence to nonweightbearing patients.^2,14^ Though SAC provide benefits to their users, they also have negative impacts including requiring twice as much energy as walking to ambulate, restricted ambulation as both arms must be used for locomotion, and can cause injuries such as brachial plexus compressive neuropathy.^10,23,24,26,32^

Additionally, SAC use can result in muscle atrophy and decreased blood flow.^11,27^ SAC require the restricted limb to be held in a static fixed position while the contralateral extremity provides forward propulsion. With unloading and decreased use of the nonweightbearing lower extremity muscles, atrophy ensues quickly and leads to a decrease in muscle size and quality.^5,22,27^ It has been studied that antigravitation muscles are especially susceptible to atrophy, and lower limb suspension leads to known decreases in muscle mass and variable decreases in strength.^5,11^ Muscle contraction in the lower extremity also contributes to heightened levels of venous return and decreased blood stasis.^3,8^ When muscle activity is decreased in a nonweightbearing lower extremity, the risk of developing a deep vein thrombosis will increase.^3,8^

**Figure 1.**
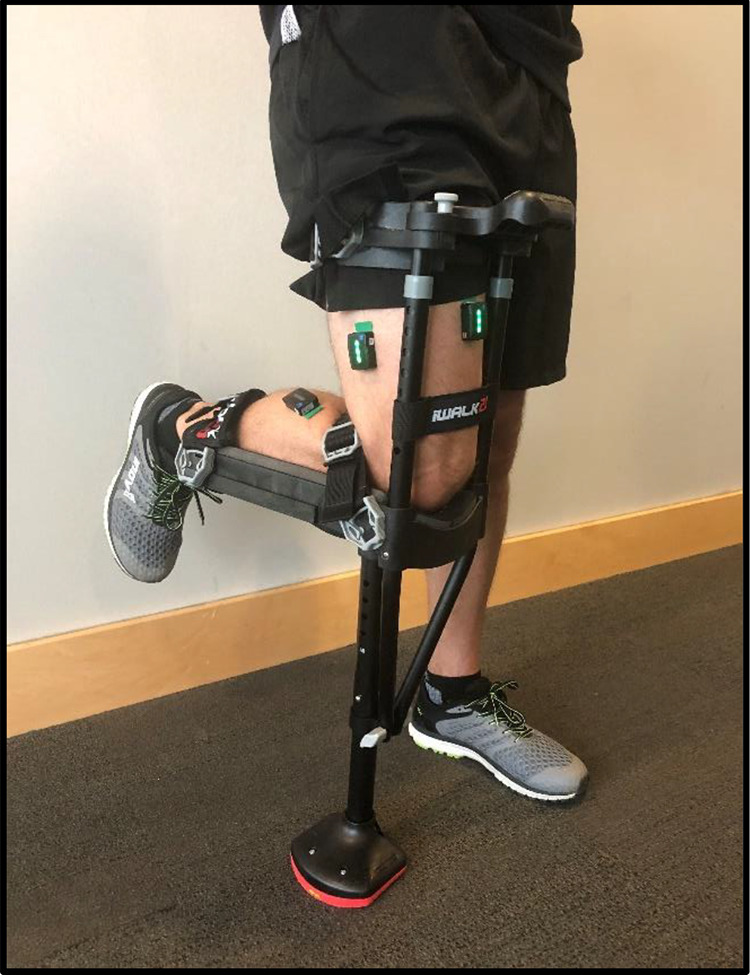
This image shows the HFSC (iWALKFree) and Trigno Avanti wireless Bluetooth sensors attached to the dominant leg. The lower part of the nonweightbearing leg is held in a horizontal position by the knee plate of the HFSC. The sensors are attached to the skin overlying the lateral gastrocnemius (left), vastus lateralis (middle), and rectus femoris (right). Gluteus maximus sensor placement cannot be seen in this image.

Recently, new ambulatory aids have been designed to allow for increased quality of life and recovery while facing nonweightbearing restrictions. Hands-free single crutch (HFSC) devices have been shown to have muscular activity that does not deviate as much from that of normal gait when compared with other ambulatory aids.^27^ AN HFSC utilizes both lower extremities for locomotion while allowing for the injured area to remain nonweightbearing. One type of HFSC is the iWALKFree (iWALKFree, Mansfield, Ontario, Canada) and a recent study found that patients prefer the iWALKFree when compared to SAC because of decreased levels of perceived exertion and discomfort.^23^ This item is widely available in retail stores and on the Internet without the need of a prescription. This HFSC operates by resting the injured area horizontally on a knee plate, while the knee and upper area of the leg support the user’s body weight during the gait cycle (Figure 1). HFSC ideally will allow for heightened levels of muscle engagement in the injured lower extremity, leading to decreased atrophy, increased blood flow, and enhanced healing.

The aim of this study was to investigate the electromyographic (EMG) muscle activity of a nonweightbearing patient while using an HFSC and SAC. Our hypothesis was that muscle activity would be similar to normal gait with increased contractility while using the hands-free single crutch compared to while using standard axillary crutches.

## Methods

The current study is a prospective comparative cohort study that used 21 healthy volunteers (3 females and 18 males) from within an active foot and ankle clinic. The sample size of 21 was based on and on par with previous studies that tested physiological muscle conductivity.^6,27-29^ Data were collected over the course of 3 days. The participants were all either active duty military or military beneficiaries with a mean age of 31.4 years (range, 20-59), mean height of 1.76 m (range, 1.57-1.91), and mean weight of 84.2 kg (range, 59.0-148.3). All the participants were right leg dominant, which was determined by asking what foot the participant would use to kick a soccer ball. Demographic data can be seen in Table 1.

**Table 1.** Demographics and Leg Dominance of Study Participants.^a^

Variable	Mean (SD); Range or n
Demographics	
Age, y	31.4 (8.8); 20-59
Height, m	1.8 (0.1); 1.6-1.9
Weight, kg	84.2 (18.2); 59.0-148.3
Body mass index	27.1 (4.4); 20.4-42.0
Sex, n	
Female	3
Male	18
Leg dominance, n	
Right	21
Left	0

Participants were included in this study if they were between the ages of 18 and 60 years and were either active duty military or a military beneficiary. Participants were excluded from this study if they had a lower extremity injury that limited their ambulatory ability, had a radiculopathy, had an injury that limited the use of their muscles for walking for 30 seconds, or if they had sustained a foot or ankle injury in the past 2 weeks.

After consenting to participating in the study, participants were fitted with SAC and an HFSC on their dominant leg by following the instructions outlined in the manufacturer’s user manual.^17^ While the HFSC was being fitted to the participant, Trigno Avanti wireless Bluetooth sensors were applied to the belly of the rectus femoris (RF), vastus lateralis (VL), lateral gastrocnemius (LG), and gluteus maximus (GM) by a board-certified orthopedic surgeon based on anatomical muscle mass. Prior to attaching the electrodes, they were calibrated and then both the sensors and the skin overlaying the belly of each muscle were cleaned with an alcohol wipe and allowed to dry. Electrode placement with the HFSC can be seen in Figure 1.

Participants were given as much time as needed to become comfortable operating the different ambulatory devices and verbal consent of being confident using those devices was obtained before proceeding with the research. No subject exceeded four minutes of practice. Participants were then instructed to walk using the HFSC at a self-selected normal speed along a 30-m walking area while 15 seconds of the gait cycle were recorded using the Delsys Trigno Wireless Electromyography system (Trigno Wireless; Delsys Inc, Boston, MA). Participants were given 5 minutes to rest and then repeated the procedure with SAC. Before ambulating with SAC, participants were instructed to maintain the nonweightbearing condition by not allowing the dominant leg to touch the ground. Twelve participants completed a third testing cycle, which included walking with no assistive device to establish a baseline control group for further comparison, whereas the other 9 participants were recorded only using the HFSC and SAC. After another 5-minute rest period, those 12 participants repeated the procedure while walking with no ambulatory assistive device.

The EMG system used for this study was the Delsys Trigno Wireless Electromyography system set to a bandwidth of 20-450 Hz, range of 11 mV, and a sampling rate of 2148 Hz. The Trigno Wireless Biofeedback System User’s Guide was followed for proper use of all EMG devices.^7^ EMG data were recorded by the EMG Acquisition Works program and then exported to the EMG Works Analysis program (Delsys, Boston, MA). The mean muscle activity and maximum voluntary isometric contraction (MVIC) were recorded for each muscle and ambulatory device combination in order to normalize the EMG data and make it largely independent of the participants and measuring devices used in this study.

Data were exported to Microsoft Office Excel (Redmond, WA) for statistical analysis. *t* tests for 2-sample means were conducted for both mean root mean square (RMS) muscle activity and the MVIC percentage for each muscle. All data was evaluated for statistical significance at the *P* <.05 level. For a given muscle, the MVIC percentage (%MVIC) was found by dividing the MVIC while using one of the ambulatory devices, by the MVIC while walking:


1 $$\text{\%}MVIC=\frac{MVIC_{i}^{j}}{MVIC_{walking}^{j}}$$

where superscript $j\in \left\{RF,GM,LG,VL\right\}$ denotes the muscles and subscript $i\in \left\{HFSC,\;SAC,Walking\right\}$ denotes the ambulatory devices. For notational simplicity, we dropped the subscript and superscript for the MVIC percentage.

## Results

**Figure 2.**
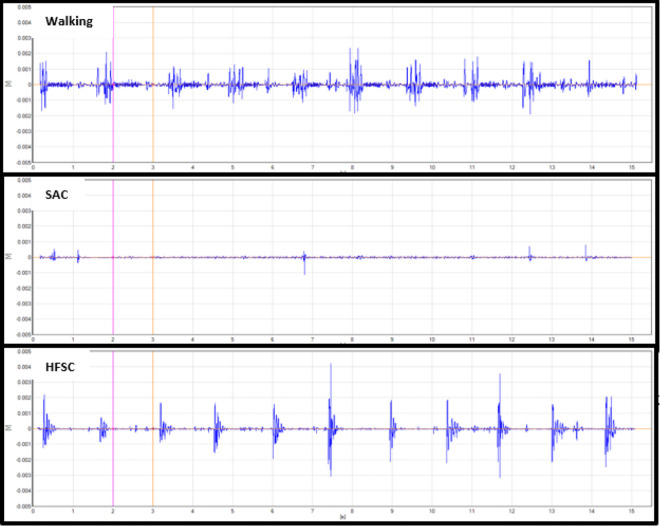
These graphs are an example of the EMG data recorded from the lateral gastrocnemius. These graphs are all from the same participant in the study and are all set to the same scale. The top graph was recorded while the participant was walking, the middle graph was while using SAC, and the bottom graph was while using the HFSC, hands-free single crutch; SAC, standard axillary crutches.

**Figure 3. fig3-2473011420939875:**
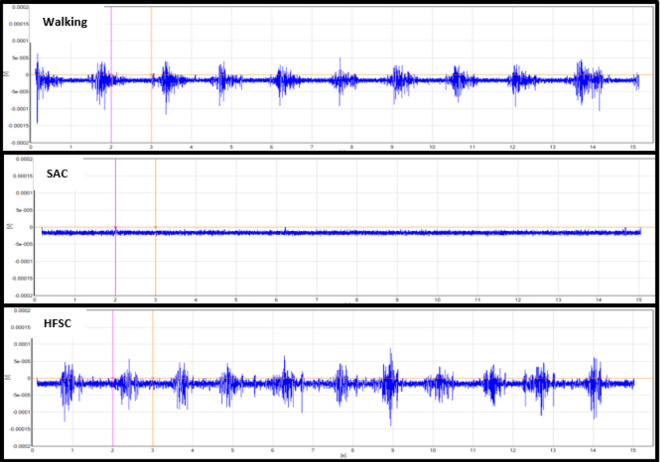
These graphs are an example of the EMG data recorded from the gluteus maximus. These graphs are all from the same participant in the study and are all set to the same scale. The top graph was recorded while the participant was walking, the middle graph was while using SAC, and the bottom graph was while using the HFSC. HFSC, hands-free single crutch; SAC, standard axillary crutches.

**Figure 4. fig4-2473011420939875:**
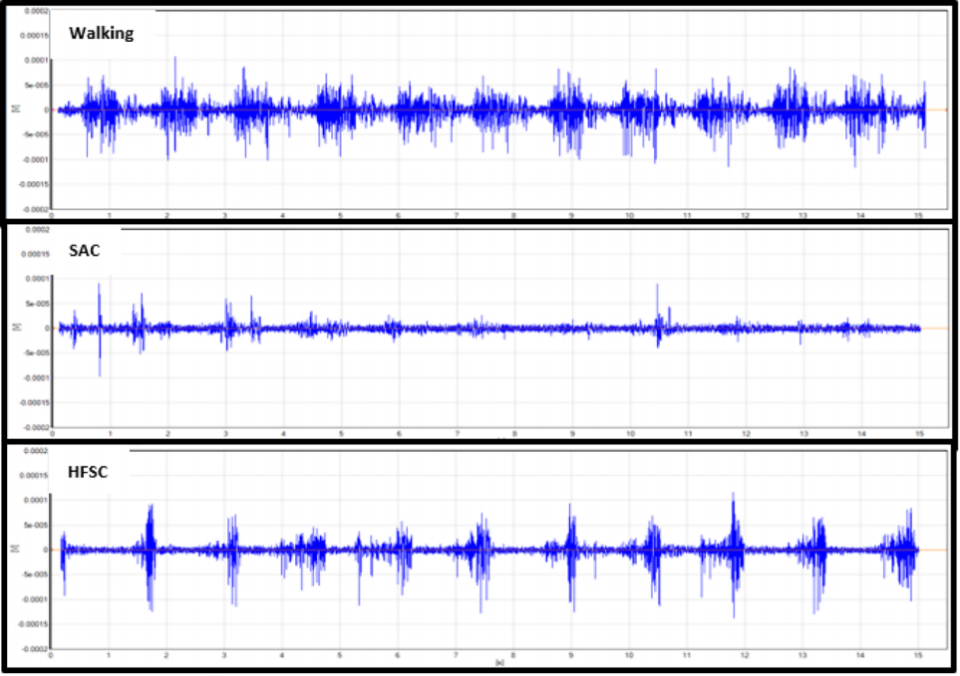
These graphs are an example of the EMG data recorded from the rectus femoris. These graphs are all from the same participant in the study and are all set to the same scale. The top graph was recorded while the participant was walking, the middle graph was while using SAC, and the bottom graph was while using the HFSC. HFSC, hands-free single crutch; SAC, standard axillary crutches.

Analysis of the mean RMS activity and MVIC percentage showed statistical differences when comparing the HFSC to SAC. The RF, GM, and LG had significantly increased levels of muscle activity while using the HFSC compared to SAC (*P* = .049, *P* = .031, *P* = .029). With the results available from this study, the VL did not demonstrate a significant increase in muscle activity while using the HFSC compared to SAC (*P* = .051). Table 2 shows the mean RMS values and standard deviation (SD) in millivolts for all 4 muscles with each ambulatory method and shows if there is a statistically significant difference in the mean muscle activity while using the HFSC compared with SAC. Some of the standard deviations in Table 2 are large because of the variance in gait pattern between participants and the amount they used each muscle for movement and balance. Figures 2 [Fig fig3-2473011420939875] [Fig fig4-2473011420939875] to 5 show examples of the EMG graphs recorded while walking, using SAC, and using the HFSC for each muscle. Finally, Figure 6 shows a side-by-side comparison of each muscle’s mean RMS muscle activity while using the HFSC and SAC, noting if the difference between the 2 is statistically significant.

**Table 2.** Mean RMS Muscle Activity.^a^

		RMS (mV)	
Muscle	Device	Mean	SD
	SAC	0.0186^b^	0.0028
GM	HFSC	0.0301^b^	0.0288
	Walking	0.0233	0.0159
	SAC	0.0304^b^	0.0071
LG	HFSC	0.1811^b^	0.3439
	Walking	0.0989	0.1503
	SAC	0.0098^b^	0.0104
RF	HFSC	0.1142^b^	0.2741
	Walking	0.1245	0.3214
	SAC	0.0412	0.1231
VL	HFSC	0.0603	0.1396
	Walking	0.0155	0.0082

Abbreviations: GM, gluteus maximus; HFSC, hands-free single crutch; LG, lateral gastrocnemius; RF, rectus femoris; RMS, root mean square; SAC, standard axillary crutches; VL, vastus lateralis.

^a^ The three types of ambulation were SAC, HFSC (iWALKFree), and walking (no assistive device).

^b^ Statistically significant values.

**Figure 5.**
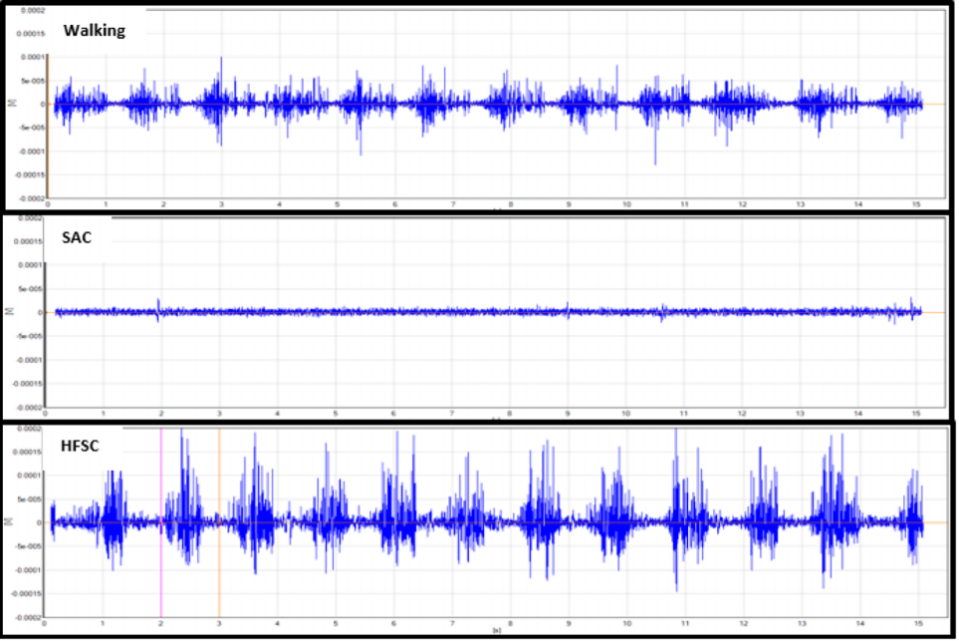
These graphs are an example of the EMG data recorded from the vastus lateralis. These graphs are all from the same participant in the study and are all set to the same scale. The top graph was recorded while the participant was walking, the middle graph was while using SAC, and the bottom graph was while using the HFSC. HFSC, hands-free single crutch; SAC, standard axillary crutches.

**Figure 6.**
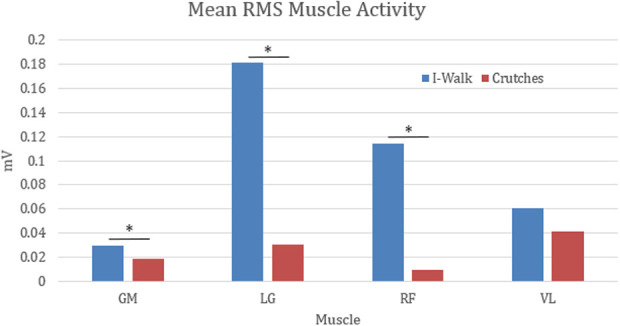
This figure shows the mean RMS muscle activity of the gluteus maximus (GM), lateral gastrocnemius (LG), rectus femoris (RF), and vastus lateralis (VL) while using the iWALKFree (HFSC) and crutches (SAC). Statistically significant differences are identified with a star. HFSC, hands-free single crutch; SAC, standard axillary crutches.

Additionally, the RF, VL, and GM all showed a statistically significant increase in MVIC percentage while using the HFSC compared to SAC (*P* = .005, *P* = .005, *P* = .013). With the results available from this study, the LG did not demonstrate a significant increase in MVIC percentage while using the HFSC compared to SAC (*P* = .076). Table 3 displays the mean MVIC percentage (%MVIC) for each muscle using each form of ambulatory aid and shows if there is a statistically significant difference in the percent MVIC while using the HFSC compared to SAC.

**Table 3.** MVIC Percentage for All Four Muscles Using Each Ambulatory Method.

Muscle	Device	% MVIC
	SAC	0.91^b^
GM	HFSC	1.97^b^
	Walking	1
	SAC	0.57
LG	HFSC	14.07
	Walking	1
	SAC	0.53^b^
RF	HFSC	3.03^b^
	Walking	1
	SAC	0.43^b^
VL	HFSC	2.51^b^
	Walking	1

Abbreviations: GM, gluteus maximus; HFSC, hands-free single crutch; LG, lateral gastrocnemius; MVIC, maximum voluntary isometriccontraction; RF, rectus femoris; RMS, root mean square; SAC, standard axillary crutches; VL, vastus lateralis.

^a^ Calculation of percentage MVIC (%MVIC) is explained in the Methods section. Walking has a percentage of 1 because its MVIC is divided by itself: (MVIC walking) / (MVIC walking) = 1.

^b^ Statistically significant values.

## Discussion

The results of our study are the first to demonstrate increased muscle recruitment and intensity while maintaining cyclic contractions consistent with bipedal gait pattern when using an HFSC compared to SAC. The RF and GM had statistically significant increases in mean muscle activity and MVIC percentage, while the LG showed a statistically significant increase in mean muscle activity and the VL showed a statistically significant increase in MVIC percentage. The heightened recruitment of these muscles while using the HFSC will potentially translate to decreased levels of muscle atrophy during the nonweightbearing period after a lower extremity injury. Reduced muscle atrophy will potentially allow for quicker rehabilitation secondary to retained balance and proprioception. The heightened cyclic muscle contractions will also facilitate vascularization of the lower extremity, while reducing potentially slowed venous return.^8^ Knee scooters hold the nonweightbearing area of the lower extremity on a platform, which has been shown to potentially contribute to deep vein thrombosis due to decreased blood flow observed via ultrasonography.^4^ Though the HFSC also holds part of the lower extremity in a horizontal position, muscle contractions were observed in the lower extremity while using the HFSC, potentially allowing for more regular levels of blood flow.

The gait cycle involves many different muscles in the body and, specifically, in the lower extremities. This study chose to examine the RF, VL, GM, and LG due to their involvement in the stance and swing phase of the gait cycle, as well as their contributions to balance and blood flow.^8,15,18,21,25^ The RF causes flexion at the hip and extension of the knee, and the VL causes extension of the knee.^15,21,25^ Both are important to balance and propulsion during both phases of the gait cycle.^15,18,21,25^ The GM is the primary extensor of the hip and is important to stability and locomotion.^15,21,25^ The LG creates plantarflexion of the foot and flexion of the knee.^15^ It is important in creating movement during the normal gait cycle, but while using the HFSC the LG is held in a static position on a knee platform.^21,25^ This study included the LG because of its impact on blood flow in the lower extremity and its effect on vascular stasis when immobilized versus when actively contracting.^8^

When reviewing the literature, other studies have used EMG to examine muscle activity while moving or exercising.^6,28,29^ Some studies have also used EMG to examine muscle activity while using ambulatory aids, such as SAC, finding that muscle inactivation can lead to atrophy.^6^ Only one other study has looked at a novel prosthesis similar to an HFSC, evaluating its muscle activity via EMG compared to other ambulatory aids.^27^ This study also found that muscle recruitment was more similar to that of walking while using the prothesis compared to the other ambulatory aids.^27^ Our paper is the first to examine muscle activity while using the iWALKFree HFSC and first to compare it to SAC muscle activity. One other study has been done on this device, noting that participants showed increased levels of preference for the HFSC over SAC due to decreased levels of perceived exertion and discomfort.^23^

Though this study has shown that the HFSC increases muscle activity while in nonweightbearing conditions, it is limited in correlating this result to decreased levels of atrophy and faster recovery timelines. Increased load on and activity of a muscle is known to lead to decreased atrophy, but many other factors affect the strength of a patient’s muscles while recovering from an injury, including nutrition, compliance, and overall healing.^23^ Furthermore, even though patients may have retained cyclic contractions while nonweightbearing, this does not directly correlate to faster rehabilitation as many other factors can influence the time it takes to regain full strength and movement in the injured lower extremity. Future studies should be done to examine the clinical outcomes of patients using these devices and examine the impact they have on the rate of recovery.

Additionally, this study is limited in its generalizability because the participants comprised mainly males and only active duty military and military beneficiaries. Future studies should be done to assess if the results of this study are reflected in the general population. This research also used the newest and best available EMG system, leading to the scarce literature available for comparing to this study. A strength of this study is that a single trained EMG technician conducted all recordings and EMG profiling. The senior author was excluded from all testing, data gathering and statistical review.

To continue investigating the HFSC, future studies should focus on learning more information about the potential risks and benefits to using such devices. More research should be done to compare the HFSC to other forms of ambulatory aid to better inform physicians of what device they should prescribe to patients facing periods of nonweightbearing after a lower extremity injury.

## Conclusion

This current study illustrates that an HFSC may provide benefits to patients facing nonweightbearing after a lower extremity injury in the form of increased muscle activation and cyclic contraction in the nonweightbearing extremity. Caution should be taken in endorsing faster recovery times, though users of the HFSC should expect increased muscle recruitment compared to using SAC. These data should be used by physicians and patients facing a decision on what type of ambulatory aid to use while nonweightbearing. The HFSC is not for every person with a lower extremity injury, but it will allow its users to maintain a bipedal gait pattern with levels of cyclic contraction that may lead to possible decreased muscle atrophy and increased blood flow.

## Supplemental Material

Supplemental Material, FAO939875-ICMJE - Comparison of Lower Extremity EMG Muscle Testing With Hands-Free Single Crutch vs Standard Axillary Crutches Click here for additional data file. Supplemental Material, FAO939875-ICMJE for Comparison of Lower Extremity EMG Muscle Testing With Hands-Free Single Crutch vs Standard Axillary Crutches by Cuyler Dewar and Kevin D. Martin in Foot & Ankle Orthopaedics
